# Phytochemical Profile and Insecticidal Potential of Two Halophytes, *Atriplex halimus* and *Suaeda fruticosa*, Against *Tribolium confusum*: In Vitro and In Silico Approaches

**DOI:** 10.1002/cbdv.71394

**Published:** 2026-06-05

**Authors:** Mounira Tabouri, Mustapha Djellouli, Khadidja Belkheir, Kamel Benotmane, Meriem Dahoun, Abdesselem Si Mohammed, Saïd Nemmiche, Hamdi Bendif, Sulaiman A. Alsalamah, Walid Elfalleh, Fehmi Boufahja, Inmaculada Moscoso‐Ruiz, Inés Cea‐Pavez, Vito Verardo, Stefania Garzoli

**Affiliations:** ^1^ Laboratory of Environment and Sustainable Development, Department of Biological Sciences, Faculty of Natural and Life Sciences Ahmed Zabana University Relizane Algeria; ^2^ Laboratory of Biochemistry, Molecular Biology and Environmental Toxicology, Faculty of Medicine Abdelhamid Ibn Badis University Mostaganem Algeria; ^3^ Department of Biological Sciences Faculty of Natural and Life Sciences Abdelhamid Ibn Badis University Mostaganem Algeria; ^4^ Department of Biology College of Science Imam Mohammad Ibn Saud Islamic University (IMSIU) Riyadh Saudi Arabia; ^5^ Center For Research and Development of Functional Food (CIDAF) Granada Spain; ^6^ Department of Nutrition and Food Science University of Granada Granada Spain; ^7^ Department of Chemistry and Technologies of Drug Sapienza University Rome Italy

**Keywords:** *Atriplex halimus*, insecticidal activity, molecular docking, phytochemical profile, *Suaeda fruticosa*, *Tribolium confusum*

## Abstract

This study aimed to investigate the phytochemical profile and evaluate the in vitro and in silico insecticidal activities of methanolic extracts from two halophytes, *Atriplex halimus* (*A. halimus*) and *Suaeda fruticosa* (*S. fruticosa*). The investigations included phytochemical screening, determination of total phenolic, flavonoid, and tannin contents, as well as compound identification by high‐performance liquid chromatography coupled with electrospray ionization quadrupole time‐of‐flight mass spectrometry (HPLC‐ESI‐QTOF‐MS). The insecticidal activity was assessed through direct‐contact bioassays against adults of *Tribolium confusum*. In addition, a molecular docking study was performed targeting the acetylcholinesterase enzyme (AChE). Phytochemical screening revealed the presence of several bioactive compounds in both plant extracts. *A. halimus* showed the highest flavonoid content, whereas *S. fruticosa* exhibited the highest levels of total phenolics and tannins, corresponding to superior insecticidal effect, as evidenced by its lower lethal concentration 50% (LC_50_) value after 96 h of treatment. HPLC‐ESI‐QTOF‐MS analysis in negative ion mode led to the identification of 24 bioactive constituents in *A. halimus* and 29 in *S. fruticosa*. The in silico study confirmed that most of the identified polyphenols exhibited binding affinity toward insect acetylcholinesterase, including kaempferol 3‐O‐rutinoside‐7‐O‐glucoside, naringin, feruloyltyramine, and rutin.

## Graphical abstract

Exploring the insecticidal activity of two Algerian halophytes, *Atriplex halimus* and *Suaeda fruticosa*.

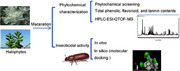

## 1 Introduction

In Algeria, cereal production in 2024 was estimated at approximately 4.1 million tonnes, close to the national average, reflecting the impact of uneven climatic conditions throughout the growing season [1]. Despite their economic importance, cereal crops remain highly vulnerable to numerous insect pests, which can cause substantial damage and yield losses [2]. Among the most destructive storage pests is *Tribolium confusum* (Duval) (Coleoptera: Tenebrionidae), commonly known as the confused flour beetle. This species infests stored cereals worldwide, proliferates rapidly on grains, and renders them unsuitable for consumption [3].

To control storage pests, chemical insecticides are widely used due to their rapid and effective action. However, intensive application raises concerns regarding human health, environmental safety, and the development of insect resistance [4]. Consequently, attention has shifted toward safer and more eco‐friendly alternatives, particularly plant‐ derived insecticides, which are recognized as promising tools for sustainable pest management [3].

The extensive saline ecosystems along Algeria's Mediterranean coast and inland regions, including sebkhas and chotts, are extreme environments where high summer evaporation leads to salt accumulation at the soil surface, severely limiting plant growth [5]. Nevertheless, halophytes have evolved remarkable physiological and biochemical adaptations, allowing them to thrive under such harsh conditions [6]. These plants are valued for diverse applications, including human nutrition, phytotherapy, animal feed, oil extraction, and fiber production [7]. Importantly, halophytes synthesize a wide range of secondary metabolites, particularly polyphenols, which are known for their insecticidal properties [8, 9].

*Atriplex halimus* L. and *Suaeda fruticosa* Forssk. Ex J.F. Gmel are two halophytic medicinal species of the *Chenopodiaceae* (currently *Amaranthaceae*) family, widely distributed across arid and semi‐arid Mediterranean regions [10, 11]. Previous studies have reported diverse biological activities for these species, including antioxidant, antimicrobial, antifungal, anti‐inflammatory, anticancer, and antidiabetic effects [12, 13].

To date, the insecticidal potential of these halophyte species in the studied region remains largely unexplored, and the available data are still limited. Therefore, the present study was designed to investigate the phytochemical profile and insecticidal potential of aqueous‐methanolic extracts of *S. fruticosa* and *A. halimus*. The phytochemical characterization included preliminary phytochemical screening and the quantification of total phenolic, flavonoid, and tannin contents. Furthermore, the identification of bioactive compounds was performed using high‐performance liquid chromatography coupled with electrospray ionization quadrupole time‐of‐flight mass spectrometry (HPLC‐ESI‐QTOF‐MS). The insecticidal activity of the extracts was evaluated against *T. confusum*. In addition, to elucidate the interaction patterns and possible mechanisms underlying the observed biological activity, the major phytochemicals tentatively identified in the extracts were further investigated through in silico molecular docking studies. This approach provides valuable insights into the potential development of natural botanical insecticides derived from halophytes.

## 2 Results and Discussion

### 2.1 Phytochemical Screening

The qualitative phytochemical analysis (Table 1) revealed that the methanolic leaf extracts of *A. halimus* and *S. fruticosa* contained a diverse range of bioactive secondary metabolites, with marked differences in their distribution and intensity. Both species tested positive for alkaloids, phenolic compounds, flavonoids, tannins, and saponins. *S. fruticosa* generally exhibited more intense reactions than *A. halimus*, particularly for phenolic compounds and tannins. Coumarins were exclusively detected in *S. fruticosa*, while glycosides were present only in *A. halimus*. Steroids were notably absent in both species. These findings are consistent with previous phytochemical characterizations as reported by [14, 15].

**TABLE 1:** Phytochemical screening of methanolic leaf extracts of *A. halimus* and *S. fruticosa*.

Phytochemical compositions	*A. halimus*	*S. fruticosa*
Alkaloids	+ ^b^	++ ^c^
Reducing sugars	−^a^	++
Glycosides	+	−
Proteins and Amino acids	+++^d^	+++
Phenolic compounds	++	+++
Flavonoids	+++	++
Tannins	++	+++
Saponins	++	+++
Steroids	−	−
Coumarins	−	+

^a^ Absence.

^b^ Low presence.

^c^ Moderate presence.

^d^ Strong presence

### 2.2 Total Phenolic, Flavonoid, and Tannin Contents

Quantitative phytochemical analysis performed on methanolic extracts showed noticeable variation among the studied species (Table 2). *S. fruticosa* was distinguished by a marked richness in total phenolic content (119.33 ± 7.24 mg GAE/g DW) and tannin content (4.51 ± 0.09 mg CE/g DW), clearly exceeding those found in *A. halimus* extract (22.23 ± 0.89 mg GAE/g and 3.53 ± 0.15 mg CE/g, respectively). In contrast, *A. halimus* exhibited a higher flavonoid concentration, reaching 73.07 ± 0.19 mg QE/g, compared with 63.92 ± 0.011 mg QE/g in *S. fruticosa*.

**TABLE 2:** Total phenolic, flavonoid, and tannin contents in methanolic extracts of the studied plant species.

	Phenolic content (mg GAE/g DW)^a^	Flavonoid content (mg QE/g DW)^b^	Tannin content (mg CE/g DW)^c^
*A. halimus*	22.23 ± 0.89^a^	73.07 ± 0.19^b^	3.53 ± 0.15^a^
*S. fruticosa*	119.33 ± 7.24^b^	63.92 ± 0.01^a^	4.51 ± 0.09^b^

Data are presented as the mean of three replicates ± standard deviation. Means followed by different letters (a and b) are significantly different with (*p* ≤ 0.05).

^a^ Milligrams of Gallic acid equivalents per gram of dry weight.

^b^ Milligrams of quercetine equivalents per gram of dry weight.

^c^ Milligrams of catechin equivalents per gram of dry weight.

In the present study, *A. halimus* extract collected from Relizane presented a higher total phenolic content than that previously reported for the same species from Bechar [15, 16], but lower than the values obtained from ethanolic leaf harvested in Sig, Mazagran, and Biskra [17]. The total flavonoid content was relatively comparable to values reported by [13, 18], whereas the total tannin content remained lower than those described in the same studies [13, 18]. For *S. fruticosa*, both total phenolic and flavonoid contents were higher than those reported in samples collected from Oran and El‐Oued (Algeria) [19, 20]. Furthermore, the tannin content reported by [19] was also lower than that obtained in the present study. The variations observed between our results and previous findings may be attributed to several factors, including genetic variability, environmental conditions such as salinity, light intensity, water availability, and soil characteristics, as well as differences in ecological and climatic conditions [16, 17]. Furthermore, harvest season, extraction methods, solvent polarity, and laboratory procedures may significantly influence both the yield and composition of extracted bioactive compounds [15].

### 2.3 Analysis of Polyphenols by HPLC–QTOF–ESI‐MS

**FIGURE 1:**
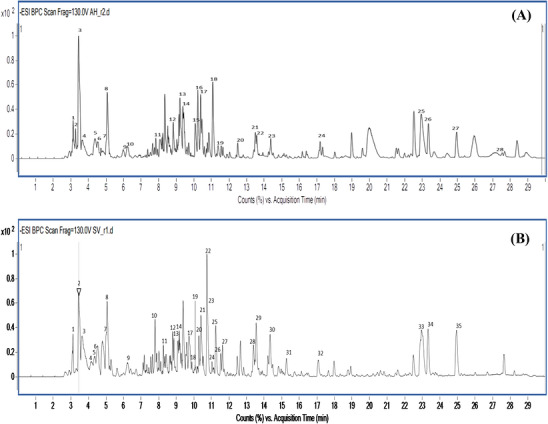
Base peak chromatograms of (A) *A. halimus* and (B) *S. fruticosa*.

Figure 1A,B and Tables 3 and 4 summarize the base peak chromatograms and the characteristics of the compounds identified in *A. halimus* and *S. fruticosa* extracts, including molecular formulae, retention times, molecular ions (*m/z*), and molecular masses. Metabolic profiling revealed a high level of chemical diversity, highlighting both shared and species‐specific metabolites. Based on QTOF–MS analysis combined with authentic standards, specialized databases (PhenolExplorer and CAS SciFinder), and previously reported literature data [21, 22], a total of 24 compounds were identified in *A. halimus* and 29 compounds in *S. fruticosa*. In addition to secondary metabolites, several primary metabolites were detected, such as sugars (glucose and D‐sedoheptulose), amino acid derivatives (N‐acetyl‐L‐phenylalanine), and lipids (palmitoleic, azelaic, malyngic, coronaric acids, and cratenacin), reflecting essential physiological and metabolic functions. Nine metabolites were common to both species, including gluconic, malic, citric, and succinic acids, pinellic, salicylic, and 17‐hydroxylinolenic acids, oxo‐tridecanoic acid sulphate, and monomethyl embelin, suggesting the presence of a shared metabolic core potentially associated with adaptation to similar environmental conditions. Despite these similarities, pronounced differences were observed in secondary metabolite composition, particularly among phenolic compounds. *S. fruticosa* exhibited a pronounced enrichment in phenolics, representing more than 50% of the identified metabolites. This abundance included several phenolic acids, such as sinapic, *p*‐coumaric, and protocatechuic acids, as well as multiple caffeic acid derivatives, notably diacetyl caffeic acid and fertaric acid.

**TABLE 3:** Identified compounds in the methanolic extract of *A. halimus* by HPLC‐ESI‐QTOF‐MS analysis.

*N*	Formula	*R*_t_ (min)	[M‐H]– (*m/z*)	Molecular mass	Compound
1	C_6_H_12_O_7_	3.122	195.0512	196.0585	Gluconic acid
2	C_6_H_12_O_6_	3.265	179.0565	180.0637	Glucose
3	C_7_H_14_O_7_	3.444	209.0671	210.0744	D‐Sedoheptulose
4	C_4_H_6_O_5_	3.663	133.0144	134.0217	Malic acid
5	C_5_H_7_NO_3_	4.368	128.0355	129.0428	Pyroglutamic acid
6	C_4_H_6_O_5_	4.519	133.0146	134.0219	Malic acid
7	C_6_H_8_O_7_	5.035	191.019	192.0269	Citric acid
8	C_5_H_7_NO_3_	5.077	128.0356	129.0428	Pyrrolidinemonocarboxylic acid
9	C_5_H_8_O_5_	5.994	147.0303	148.0375	Citramalic acid
10	C_4_H_6_O_4_	6.229	117.0195	118.0268	Succinic acid
11	C_13_H_16_O_9_	7.821	315.0726	316.0799	Protocatechuic acid 4‐glucoside
12	C_13_H_24_O_7_S	8.812	323.1173	324.1246	2‐Octyl‐2‐sulfopentanedioic acid
13	C_9_H_8_O_7_S	9.197	258.9921	259.9995	Caffeic acid 3‐sulfate^a^
14	C_9_H_8_O_7_S	9.387	258.9923	259.9996	Caffeic acid 3‐sulfate^a^
15	C_7_H_6_O_4_	10.063	153.0198	154.0271	Dihydroxybenzoic acids
16	C_6_H_6_O_6_	10.2	173.0094	174.0166	Trans‐aconitic acid
17	C_15_H_14_O_9_	10.342	337.0569	338.0641	Quercetin dihydrate^a^
18	C_13_H_24_O_6_S	11.048	307.1227	308.13	Oxo‐tridecanoic acid sulphate
19	C_8_H_8_O_5_S	11.354	215.0022	216.0095	Alpha‐sulfophenylacetic acid
20	C_18_H_19_NO_4_	12.469	312.1246	313.1318	Feruloyltyramine
21	C_7_H_6_O_3_	13.474	137.0246	138.0318	Salicylic acid
22	C_6_H_6_O	13.476	93.0348	94.0421	Phenol
23	C_18_H_34_O_5_	14.35	329.2336	330.2409	Pinellic acid
24	C_18_H_28_O_4_	17.116	307.1921	308.1993	Monomethyl ambelin
25	C_18_H_30_O_3_	22.979	293.2127	294.22	17‐Hydroxylinolenic acid
26	C_18_H_30_O_3_		293.2127	294.22	17‐Hydroxylinolenic acid
27	C_18_H_32_O_3_	24.94	295.228	296.2353	Coronaric acid
28	C_16_H_30_O_2_	27.556	253.2173	254.2246	Palmitoleic acid

All identified compounds showed mass errors lower than 5 ppm (<5 ppm).

^a^ Confirmed using authentic reference standards.

**TABLE 4:** Identified compounds in the methanolic extract of *S. fruticosa* by HPLC‐ESI‐QTOF‐MS analysis.

*N*	Molecular formula	*R*_t_ (min)	[M‐H]– (*m/z*)	Molecular mass	Compound
1	C_6_H_12_O_7_	3.113	195.0516	196.0589	Gluconic acid
2	C_11_H_12_O_5_	3.458	223.046	224.0534	Sinapic acid
3	C_4_ H_6_O_5_	3.657	133.0148	134.0221	Malic acid
4	C_6_ H_8_O_7_	4.177	191.02	192.0273	Citric acid
5	C_5_H_7_NO_3_	4.355	128.0357	129.043	L‐Pyroglutamic acid
6	C_4_H_4_O_4_	4.523	115.0042	116.0115	Fumaric acid
7	C_6_ H_8_ O_7_	5.025	191.0201	192.0273	Citric acid
8	C_5_ H_7_NO_3_	5.072	128.0356	129.0424	L‐Pyroglutamic Acid
9	C_4_H_6_O_4_	6.224	117.0194	118.0266	Succinic acid
10	C_14_ H_20_ O_8_	7.802	315.1086	316.1159	Vanilloloside
11	C_14_H_14_O_9_	8.426	325.0566	326.064	Fertaric acid
12	C_33_H_40_O_20_	9.091	755.206	756.2133	Kaempferol 3‐O‐rutinoside 7‐O‐glucoside
13	C_16_H_18_O_10_	9.288	369.0834	370.0907	Fraxin
14	C_26_H_28_O_16_	9.346	595.1308	596.1382	Quercetin 3‐O sambubioside^a^
15	C_12_H_12_O_4_	9.392	219.0668	220.074	Eugenitin
16	C_13_H_12_O_6_	9.395	263.0563	264.0636	Diacetyl caffeic acid
17	C_27_H_30_O_16_	9.741	609.1472	610.1545	Rutin
18	C_13_H_12_O_6_	9.89	263.0566	264.0637	Diacetyl caffeic acid
19	C_7_H_6_O_4_	10.103	153.0198	154.027	Protocatechuic Acid
20	C_22_H_22_O_12_	10.361	477.1045	478.1118	isorhamnetin‐3‐O‐glucoside^a^
21	C_11_H_13_NO_3_	10.414	206.0825	207.0899	N‐Acetyl‐L‐phenylalanine
22	C_27_H_32_O_14_	10.748	579.1742	580.1816	Naringin
23	C_9_H_8_O_3_	10.867	163.0401	164.0474	Coumaric acid
24	C_13_H_12_O_6_	11.141	263.0566	264.0639	Diacetyl caffeic acid
25	C_13_H_24_O_6_S	11.251	307.122	308.13	Oxo‐tridecanoic acid sulphate
26	C_9_H_16_O_4_	11.534	187.0977	188.105	Azelaic acid
27	C_29_H_32_O_15_	11.628	619.1677	620.175	Cratenacin
28	C_7_H_6_O_3_	13.476	137.0245	138.0318	Salicylic acid
29	C_18_H_32_O_5_	13.548	327.218	328.2253	Malyngic acid
30	C_18_H_34_O_5_	14.34	329.2336	330.241	Pinellic acid
31	C_16_H_12_O_7_	15.27	315.0513	316.0586	Isorhamnetin^a^
32	C_18_H_28_O_4_	17.081	307.1919	308.1993	Monomethyl embelin
33	C_18_H_30_O_3_	22.986	293.2124	294.2197	17‐Hydroxylinolenic acid
34	C_18_H_30_O_3_	23.332	293.2124	294.2197	17‐Hydroxylinolenic acid
35	C_18_H_32_O_3_	24.948	295.2279	296.2352	13‐Hydroxyoctadecadienoic Acid

All identified compounds showed mass errors lower than 5 ppm (<5 ppm).

^a^ Confirmed using authentic reference standards.

In addition, kaempferol and quercetin were mainly detected in glycosylated forms (rutin, quercetin 3‐O‐sambubioside, kaempferol 3‐O‐rutinoside‐7‐O‐glucoside, and isorhamnetin‐3‐O‐glucoside), along with the flavanone naringenin, further highlighting the flavonoid richness of the extract. These findings differ from those reported by [20], who identified gallic acid, vanillic acid, caffeic acid, p‐coumaric acid, vanillin, rutin, and quercetin in extracts of the same species. Such differences may be attributed to variations in environmental conditions, genetic diversity among plant populations, and metabolic differences associated with species adaptation within the *Chenopodiaceae* family [20].

In contrast, *A. halimus* exhibited a comparatively less diverse phenolic profile, mainly characterized by caffeic acid 3‐sulfate, dihydroxybenzoic acid derivatives, and protocatechuic acid 4‐glucoside. Hydrated quercetin was identified as the predominant flavonoid in this species. These observations are consistent with previous reports [23], supporting the hypothesis that *A. halimus* possesses a distinct secondary metabolite pattern compared with *S. fruticosa*.

The presence of kaempferol, rutin, and quercetin in our samples is of particular interest, given their documented insecticidal properties. These compounds have been reported to interfere with insect development and feeding behavior, especially in the control of insect larvae, which represent the most destructive stage for crops [24].

### 2.4 Insecticidal Activity

**FIGURE 2:**
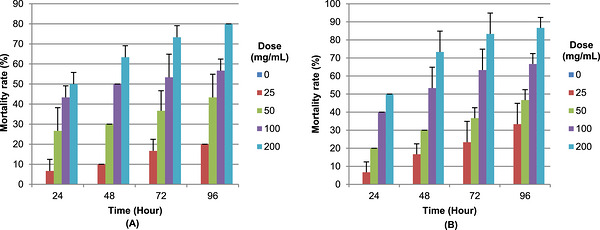
Evaluation of adult *T. confusum* mortality rate exposed to different concentrations of methanolic extracts (A) *A. halimus* and (B) *S. fruticosa*.

Figure 2A,B (A,B) illustrates the temporal evolution of mortality in adult populations of *T. confusum* exposed to different concentrations of hydromethanolic plant extracts. The results showed that both plant extracts induced significantly higher mortality rates compared with the control treatment. The highest mortality levels were recorded after 96 h of exposure at 200 mg/mL, reaching 86.66 ± 5.77% and 80.00 ± 00% for *S. fruticosa* and *A. halimus* extracts, respectively. In contrast, minimal mortality (6.66 ± 5.77 %) was observed after 24 h at the lowest tested concentration (25 mg/mL), indicating limited acute toxicity during short exposure periods.

Each column represents the mean of three replicates, and error bars indicate the standard deviation. No mortality was observed in the control group (0%).

On the other hand, LC_50_ values (Table 5) decreased progressively with increasing exposure time. After 24 h, relatively high LC_50_ values were recorded for both extracts, reaching 169.66 ± 8.85 mg/mL for *Suaeda fruticosa* and 164.75 ± 26.22 mg/mL for *A. halimus*. After 96 h of exposure, these values markedly decreased to 84.73 ± 0.00 mg/mL and 50.03 ± 12.65 mg/mL, respectively. At intermediate exposure times, *S. fruticosa* consistently exhibited lower LC_50_ values (108.12 ± 16.004 mg/mL at 48 h; 82.72 ± 2.36 mg/mL at 72 h), compared with *A. halimus* (116.49 ± 18.77 mg/mL at 48 h; 89.63 ± 21.91 mg/mL at 72 h). The consistently lower LC_50_ values observed for *S. fruticosa* indicate a higher overall insecticidal potency relative to *A. halimus*. This enhanced activity is likely due to its higher content of phenolic compounds and flavonoids, as revealed by phytochemical profiling.

**TABLE 5:** LC_50_ values of plant extracts against *T. confusum* adults at four times intervals.

	LC_50_^1^ (mg/mL)	
Time	*A. halimus*	*S. fruticosa*
24 h	164.75 ± 26.22	169.66 ± 8.85
48 h	116.49 ± 18.77	108.12 ± 16.004
72 h	89.63 ± 21.91	82.72 ± 2.36
96 h	84.73 ± 00	50.03 ± 12.65

Data are presented as the mean ± standard deviation of three replicates with (*p* ≤ 0.05).

^a^ Median lethal concentration.

The insecticidal activity observed in the present study is consistent with previous reports, highlighting the potential of these species as natural bioinsecticides. For instance, [25] demonstrated the efficacy of a 5% hydromethanolic extract of *A. halimus* against *Dactylopius opuntiae* adults, achieving a mortality rate of 90.00 ± 10% after 8 days of exposure, with an estimated LC_50_ of 2.10 mg/mL. Similarly, [26] reported significant larvicidal and adulticidal activities of the methanolic extract of *S. fruticosa* against the Mediterranean fruit fly (*Ceratitis capitata*) after 24 h of treatment, with LC_50_ values of 7.1 mg/g of food for larvae and 0.034 g/mL for adults. A comparative study by [8] further demonstrated that the application of *A. halimus* and *S. fruticosa* extracts prepared using different solvents allows an excellent control of the Cotton Aphid (*Aphis gossypii)*, emphasizing that solvent selection strongly influences the insecticidal activity, as reflected in LC_50_ values variations under similar experimental conditions. Although LC_50_ values reported in previous studies differ from those obtained in the present work, such discrepancies may be explained by differences in insect species, exposure duration, experimental conditions, bioassay methods, and variations in extract chemical composition.

### 2.5 Docking Results

To correlate the experimental results of the phytochemical study and the inhibitory activity observed against *T. confusum* of molecules already identified by HPLC‐ESI‐QTOF in the extracts, docking constitutes a very reliable tool to predict the binding free energy and evaluate the affinity of this molecule with its biological target.

As depicted in Tables 6 and 7, molecular docking studies performed on extracts of *A. halimus* and *S. fruticosa* revealed a variable negative charge affinity of selected phenolic and flavonoid compounds for the target protein, ranging from −10.2 to −5.0 kcat/mol, reflected by their binding energies. All RMSD values were < 2.0 Å, indicating good agreement between docked and crystallographic poses.

**TABLE 6:** Binding free energy values Amino acids and types of interactions involved in effectors and ligands binding calculated through the molecular docking of the major polyphenols identified in *A.halimus*.

Compounds	Binding energies (kcat/mol)	Amino acids and interaction types	Distance (Å)
Feruloyltyramine	−9.5	TYR A: 71: Conventional hydrogen bond	2.39
Quercetin dihydrate	−8.3	ASP A: 375: Pi–donor hydrogen Bond	2.50
		TYR A: 324: Pi–donor hydrogen Bond	2.90
Caffeic acid 3‐sulfate	−7.2	TYR A: 324: Pi–donor Hydrogen Bond	2.92
Protocatechuic acid 4‐glucoside	−6.6	ARG A: 259: Pi–Alkyl	5.08
Dihydroxybenzoic acid	−6.3	PHE A: 371: Pi–Pi T‐shaped	5.08
Salicylic acid	−6.0	TRP: 83 A: Pi–Pi T‐shaped	5.36
		TRP: 83 A: Pi–Pi T‐shaped	5.49
		GLY: 150 A: Pi–sigma	3.75
		TYR: 370 A: Pi–Pi T‐shaped	5.31
Phenol	−5.0	TYR: 374 A: Pi–Pi stacked	4.91

RMSD values < 2.0 Å.

**TABLE 7:** Binding free energy values Amino acids and types of interactions involved in effectors and ligands binding calculated through the molecular docking of the major polyphenols identified in *S.fruticosa*

Compounds	Binding energies (kcat/mol)	Amino acids and interaction types	Distance (Å)
Kaempferol 3−O−rutinoside 7−O−glucoside	−10.2	TRP A:321: PiPi stacked	4.67
		TRP A:321: PiPi stacked	4.72
		TRP A:321: PiPi stacked	5.1
		TRP A:321: PiPi stacked	5.28
Naringin	−9.5	VAL A: 318: Pi–alkyl	4.08
Rutin	−8.7	ASP A: 375: Carbon–hydrogen bond	3.32
		TYR A: 73: Pi–sigma	3.61
		TYR A: 73: Pi–alkyl	5.49
Coumaric acid	−8.4	TYR A: 324: Pi–donor Hydrogen Bond	2.88
Fertaric acid	−6.4	THR A: 469: Conventional hydrogen bond	2.71
		GLU A: 485: Carbon–hydrogen bond	3.54
Isorhamnetin	−8.5	TYR A: 324: Pi–Donor Hydrogen Bond	2.87
Isorhamnetin−3−O−glucoside	−8.3	TRP A: 321: PiPi Stacked	4.19
		TRP A: 321: PiPi Stacked	4.52
		TRP A: 321: PiPi Stacked	4.33
		TRP A: 321: PiPi Stacked	4.89
		TYR A: 73: PiPi Stacked	5.99
Quercetin 3−O−sambubioside	−8.2	TYR A: 71: PiPi Stacked	5.67
		TYR A: 324: PiPi Stacked	4.92
		TRP A: 321: PiPi‐shaped	4.31
		TRP A: 321: PiPi‐shaped	4.43
		TRP A: 321: PiPi‐shaped	4.69
		TRP A: 321: PiPi‐shaped	4.94
Cratenacin	−8.1	XTYR A: 71: Pi‐Donor hydrogen bond	2.60
		TRP A: 321: PiPi‐shaped	5.11
		TRP A: 321: PiPi stacked	3.86
		TRP A: 321: PiPi stacked	4.33
		TRP A: 321: PiPi‐shaped	4.63
		TYR A: 324: PiPi‐shaped	4.67
Fraxin	−6.9	ASP A: 356: Conventional hydrogen bond	1.97
		ARG A: 259: Conventional hydrogen bond	3.08
		ASP A: 358: carbon–hydrogen Bond	3.46
Eugenitin	−6.3	TRP A: 83: Pi–sigma	3.92
		TRP A: 83: PiPi‐shaped	5.92
		GLY A: 150: Pi–sigma	3.51
		SER A: 238: Conventional hydrogen bond	1.94
		TRP A: 271: Pi–alkyl	4.01
		TRP A: 271: Pi–alkyl	4.39
		PHE A: 330: Pi–alkyl	4.79
		PHE A: 371: PiPi‐shaped	4.74
		PHE A: 440: Pi–alkyl	5.29
		TYR A: 37: PiPi‐shaped	5.54
		TYR A: 370: Pi–alkyl	5.23
Sinapic acid	−6.1	TRP A: 321: PiPi stacked	4.02
		TRP A: 321: PiPi stacked	4.32
Diacetyl caffeic acid	−6.0	ILE A: 82: Pi–sigma	3.50
		LEU A: 471: Pi–alkyl	5.23
Salicylic acid	−6.0	TRP A: 83: Pi–Pi T‐shaped	5.36
		TRP A: 83: Pi–Pi T‐shaped	5.49
		GLY A: 150: Pi–sigma	3.75
		TYR A: 370: Pi–Pi T‐shaped	5.31

RMSD values < 2.0 Å.

**FIGURE 3:**
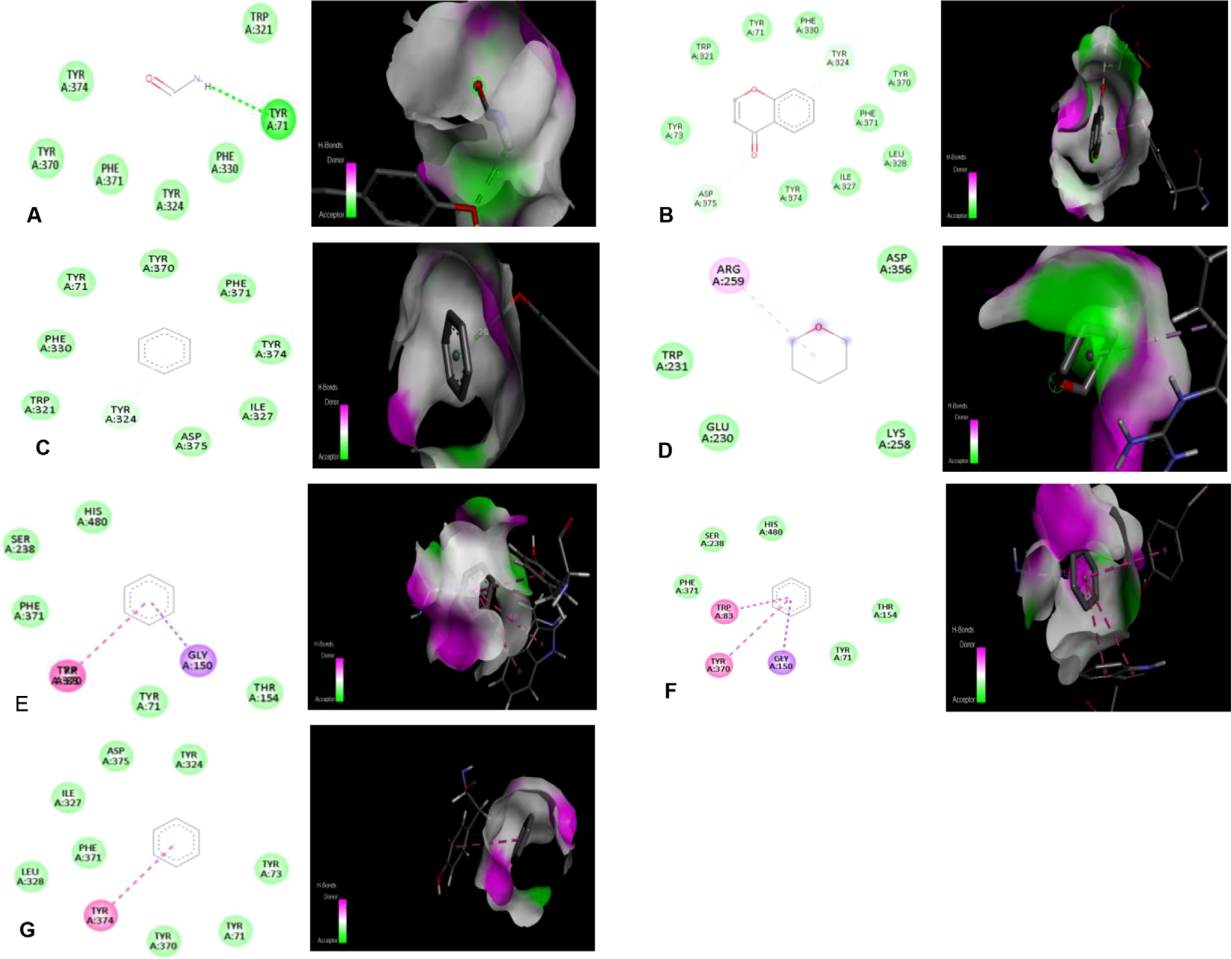
Intermolecular interaction of the 2D (left) and the 3D (right) structures of acetylcholinesterase (AChE) protein with identified compounds of *A. halmus* extract(A) feruloyltyramine, (B) quercetin dihydrate, (C) caffeic acid 3‐sulfate, (D) protocatechuic acid 4‐glucoside, (E) dihydroxybenzoic acid, (F) salicylic acid, and (G) phenol.

**FIGURE 4:**
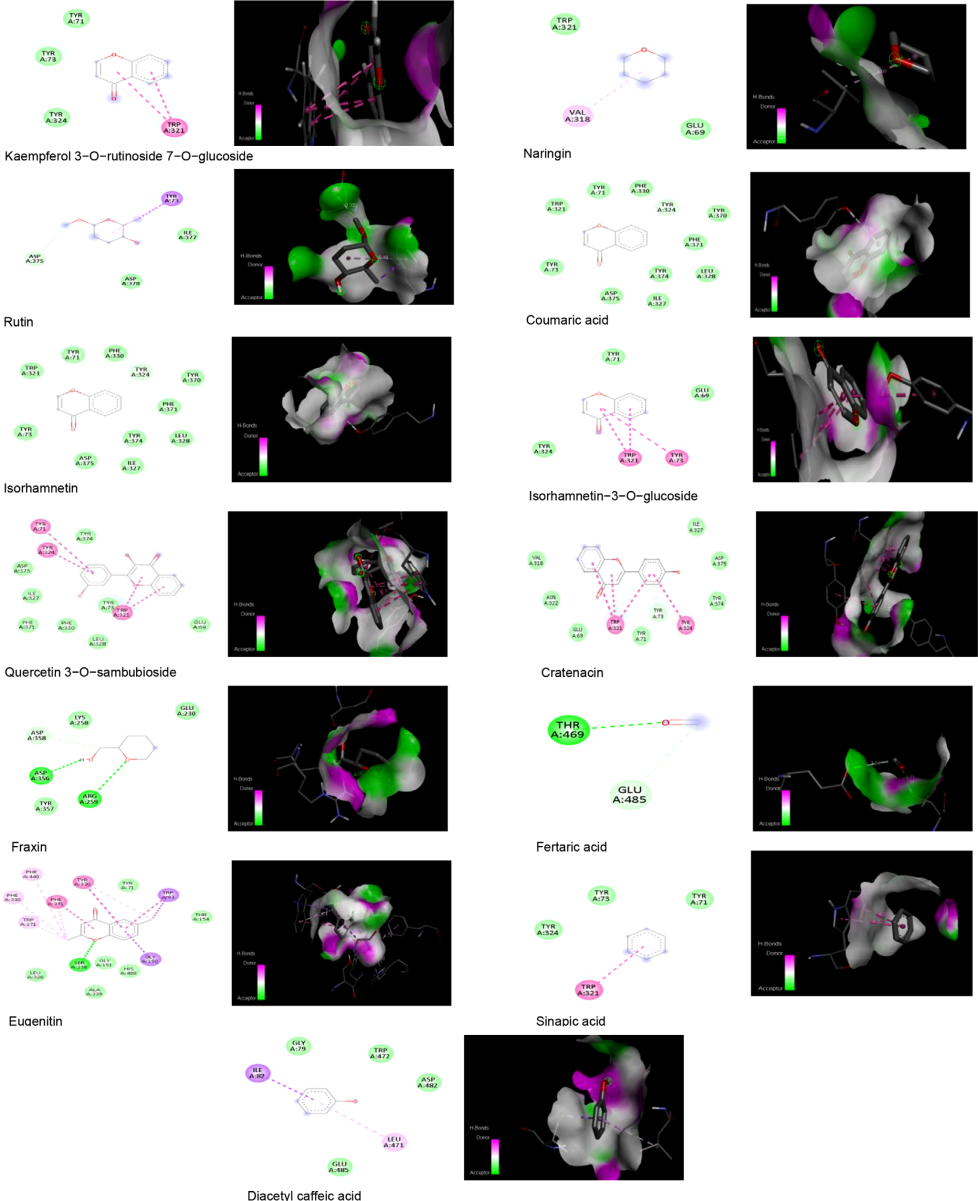
Intermolecular interaction of the 2D (left) and the 3D (right) structures of acetylcholinesterase (AChE) protein with identified compounds of *S.fruticosa* extract.

For *A. halimus*, feruloyltyramine (−9.5 kcal/mol) and quercetin dihydrate (−8.3 kcal/mol) showed the highest affinities with AChE, forming classical and Pi–donor hydrogen bonds with key amino acids such as TYR71, ASP375, and TYR324, with optimal distances of 2.39–2.92 Å, suggesting stable and specific interactions (Figure 3). In the case of *S. fruticosa* (Figure 4), Kaempferol 3‐O‐rutinoside 7‐O‐glucoside exhibited the highest binding energy (−10.2 kcal/mol), primarily via stacked Pi–Pi interactions with TRP321, while other flavonoids such as Naringin (−9.5 kcal/mol) and Rutin (−8.7 kcal/mol) showed Pi–alkyl and Pi–sigma interactions with VAL318, TYR73, and ASP375. Phenolic acids such as coumaric acid and Isorhamnetin also contributed via Pi–donor and conventional hydrogen bonds at short distances (∼2.8–2.9 Å), while smaller compounds like salicylic acid and sinapic acid interacted mainly via Pi–Pi or Pi–sigma interactions at longer distances (3.75–5.49 Å), indicating moderate affinity. Overall, these results suggest that glycosylated flavonoids and certain phenolic acids from both species have significant potential to modulate the Acetylcholinesterase (AChE) via stable interactions, with *S.fruticosa* compounds showing superiority in terms of maximum binding energy, while *A. halimus* shows particularly strong hydrogen interactions. These findings also support the results observed in in vitro studies.

## 3 Conclusions

This study demonstrates that the methanol extracts of *S. fruticosa* and *A. halimus* contain considerable amounts of total phenolics, flavonoids, and tannins, and exhibit notable insecticidal activity. HPLC–ESI–QTOF–MS profiling enabled the identification of several important secondary metabolites. Both extracts showed moderate cytotoxicity against *T. confusum*. Furthermore, molecular docking studies confirmed that selected compounds can effectively inhibit AChE, a key enzyme in insect neurotransmission, and detailed their interactions with the enzyme active site. These findings highlight the potential of these halophytic plants as eco‐friendly alternatives to synthetic pesticides and support their possible application in sustainable pest management strategies.

## 4 Experimental Section

### 4.1 Plant Material Collection

The aerial parts of *A. halimus* and *S. fruticosa* were collected in January 2024 from natural saline habitats located in northwestern Algeria. *A. halimus* was harvested from Belassel (Relizane, Algeria; 35°44′12.7″N, 0°32′23.9″E), characterized by a soil pH of 8.1 and salinity of 12.48 dS/m. *S. fruticosa* was collected from Sebkha (Oran, Algeria; 35°36′11.5″N, 0°41′16.2″W), with a soil pH of 7.4 and salinity of 21.15 dS/m. The plant material was identified in the Department of Biology, Oran 1 University, Algeria, by Dr.Yamina HALFAOUI, using standard taxonomic keys [27]. Voucher specimens were deposited under the numbers RE‐2024‐01 (*A. halimus*) and RE‐2024‐02 (*S. fruticosa*) at the herbarium of the Faculty of Natural and Life Sciences, University of Relizane, Algeria. The collected leaves were washed and air‐dried at room temperature away from direct sunlight. The dried plant material was then finely ground into a uniform powder using an electric grinder. The resulting powder was stored in glass jars until further use.

### 4.2 Insect Collection and Rearing

Adult of *T. confusum* were obtained from a stock of infested soft wheat grains collected in the Sidi M'hamed Benali area (Relizane, Algeria) and reared in glass jars containing wheat flour as a food substrate. Each jar was covered with a fine mesh screen to ensure adequate ventilation and prevent insect escape. The cultures were maintained at 28 ± 2°C and 70% relative humidity, following the protocol described by [28].

### 4.3 Extracts Preparation

A total of 2.5 g of plant powder was mixed with 100 mL of methanol–water solution (75:25, v/v) under continuous stirring at room temperature for 24 h. After maceration, the mixtures were filtered through Whatman filter paper N°:1, and the filtrates were concentrated under reduced pressure using a rotary evaporator (LabTech‐ EV400) at 40°C.

### 4.4 Qualitative Phytochemical Screening

Preliminary phytochemical screening of the methanolic plant extracts was conducted to detect major classes of phytoconstituents, following standard procedures described by [29, 30].

### 4.5 Detection of Alkaloids

Alkaloids were tested by adding a few drops of 2% aqueous picric acid solution to 1 mL of plant extract. The formation of a yellow to yellow–orange precipitate indicated the presence of alkaloids.

### 4.6 Detection of Reducing Sugars

Reducing sugars were detected by mixing 1 mL of each extract with equal volumes of Fehling's A and B solutions, followed by heating in a water bath for 10 min. A red precipitate indicated their presence.

### 4.7 Detection of Glycosides

Glycosides were identified by adding 400 µL of glacial acetic acid, one drop of 5% FeCl_3_, and concentrated H_2_SO_4_ along the sides of the test tube to 1 mL of extract. The formation of a brown ring indicated a positive result.

### 4.8 Detection of Proteins and Amino Acids

Proteins and amino acids were revealed by adding a drop of saturated ninhydrin solution in acetone to the extract, followed by heating. The appearance of a purple color indicated their presence.

### 4.9 Detection of Phenolic Compounds

Phenolic compounds were detected by adding 1–2 drops of 5% aqueous ferric chloride solution (FeCl_3_) to 1 mL of extract. A blue–black or green–black coloration indicated their presence.

### 4.10 Detection of Flavonoids

Flavonoids were identified using the Shinoda test, in which a few fragments of magnesium ribbon were added to 1 mL of extract, followed by the dropwise addition of concentrated HCl. The formation of a red‐to‐pink color indicated the presence of flavonoids.

### 4.11 Detection of Tannins

Tannins were determined using the Braymer test, where distilled water and three drops of 10% ferric chloride solution were added to 1 mL of extract. A blue–green coloration indicated the presence of tannins.

### 4.12 Detection of Saponins

Saponins were identified using the foam test, in which 2 mL of extract was vigorously shaken with 5 mL of water. The formation of a stable foam persisting for more than 10 min indicated the presence of saponins.

### 4.13 Detection of Steroids

Steroids were detected using the Salkowski test. One milliliter of extract was treated with 2 mL of chloroform, followed by careful addition of 1 mL of concentrated H_2_SO_4_ along the sides of the test tube. A red or reddish‐brown color in the lower layer indicated a positive result.

### 4.14 Detection of Coumarins

Coumarins were confirmed using the NaOH test, in which a few drops of 10% sodium hydroxide solution were added to 1 mL of extract. A yellow color indicated the presence of coumarins.

### 4.15 Total Phenolic Content

The total phenolic content of plant extracts was determined using the Folin‐Ciocalteu method according to [31], with some modifications. Briefly, 20 µL of each sample was mixed with 1.58 mL of distilled water and 100 µL of Folin–Ciocalteu reagent. The mixture was incubated in the dark for 1–8 min at room temperature, followed by the addition of 300 µL of 20% sodium carbonate solution. After incubation for 2 h, absorbance was measured at 760 nm using a UV–vis spectrophotometer (Dynamica HALO XB‐10). The total phenolic content was calculated using a Gallic acid calibration curve (0.025–1 mg/mL) and expressed as milligrams of gallic acid equivalent per gram of dry weight (mg GAE/g DW).

### 4.16 Total Flavonoid Content

The total flavonoid content was estimated according to the method described by [31], using the aluminum chloride colorimetric assay.1 mL of plant extract was mixed with 1 mL of 2% methanolic AlCl_3_ solution and incubated for 30 min at room temperature. The absorbance was measured at 415 nm against using methanol as a blank. Flavonoid concentrations were calculated from a quercetin calibration curve and expressed as milligrams of quercetin equivalent per gram of dry extract (mg QE/g).

### 4.17 Total Tannin Content

The total tannin content was determined by the vanillin reagent method as described by [32]. Briefly, 250 µL of each extract was mixed with 1.5 mL of 4% vanillin solution (prepared in methanol) and 750 µL of concentrated hydrochloric acid (HCl). The mixture was incubated at room temperature for 15 min, and absorbance was measured at 500 nm against a blank. Tannin concentrations were calculated from a catechin calibration curve and expressed as milligrams of catechin equivalents per gram of dry weight (mg CE/g DW).

### 4.18 HPLC‐ESI‐QTOF‐MS Analysis

Chromatographic analyses were performed using an Agilent 1260 HPLC system (Agilent Technologies, Palo Alto, CA, USA) equipped with a Zorbax Eclipse Plus RP‐C18 column (150 mm × 4.6 mm i.d., 1.8 µm particle size). The HPLC system was coupled to a Q‐TOF mass spectrometer with an ESI Jet Stream interface (model 6540, Agilent Technologies), operating in negative‐ion mode over a mass range of 50–1700 *m/z*. Data acquisition and processing were carried out using MassHunter software (version B.06.00, Agilent Technologies). The mobile phase consisted of water containing 0.1% formic acid (solvent A) and acetonitrile (solvent B), using a gradient program as follows: 5% B at 0 min, 35% B at 5 min, 85% B at 22 min, and 95% B at 24 min, followed by re‐equilibration to 5% B at 25 min and maintained for 5 min, resulting in a total run time of 30 min. The flow rate was set at 0.5 mL/min, the injection volume was 10 µL, and the column temperature was maintained at 25°C. Samples were kept at 4°C during analysis. The mass spectrometer parameters were set as follows: capillary voltage 4000 V, nebulizer pressure 320 psig, fragmentor voltage 130 V, nozzle voltage 500 V, skimmer voltage 45 V, and octopole voltage 750 V. The instrument was calibrated and tuned according to the manufacturer's instructions, with continuous internal mass calibration using reference ions at *m/z* 112.985587 (trifluoroacetate) and 1033.988109 (hexakis(1H,1H,3H‐tetrafluoropropoxy) phosphazine adduct). Major compounds were detected using molecular feature extraction, filtered against solvent blanks with a relative abundance threshold of 0.2%, and tentatively identified by comparison with phenolic compound databases (Phenol‐Explorer, CAS SciFinder) and relevant literature [21, 22]. All detected compounds showed mass errors lower than 5 ppm. In addition, selected compounds, including caffeic acid, quercetin, and isorhamnetin, were confirmed using authentic reference standards analyzed under identical chromatographic conditions by comparing their retention times and accurate mass spectra.

### 4.19 Evaluation of Insecticidal Activity

The direct contact insecticidal activity of methanolic extracts from halophytic plants against *T. confusum* was performed according to the method described by [33] with some modifications. Four extract concentrations (25, 50, 100, and 200 mg/mL) were prepared using a 2% Tween solution as a dispersing agent. A volume of 0.5 mL of each concentration was applied using a hand‐held sprayer on ten adult insects. The treated insects were subsequently transferred to Petri dishes containing filter paper matching the diameter of the dishes. The negative control consisted of 2% Tween solution alone. Aeration was ensured thought small holes in the dish lids. Insect mortality was recorded after 24, 48, 72, and 96 h of exposure.

### 4.20 Estimation of Mortality Rate and Median Lethal Concentration (LC_50_)

Observed mortality rates were corrected using the formula of [34]

$$\begin{array}{ccc} \mathrm{Mortality}\;\mathrm{rate}\left(\%\right) & = & \frac{\left(\%\;\mathrm{test}\;\mathrm{mortality}\;-\;\%\;\mathrm{Control}\;\mathrm{mortality}\right)}{100\;-\;\%\;\mathrm{Control}\;\mathrm{mortality}\;}\\ & & \times 100. \end{array}$$

If control mortality exceeded 20%, the test was repeated.

The median lethal concentration (LC_50_) for each plant extract was determined using log‐Probit regression analysis according to [35].

### 4.21 In Silico Studies

#### 4.21.1 Protein Preparation

The enzyme of acetylcholinesterase (PDB ID: 6XYS) [36] was obtained from the RCSB Protein Data Bank and saved in PDB file format. The protein structure was prepared by removing water molecules and bound ligands from the three‐dimensional (3D) structure using Discovery Studio 2025 Client. Protons and charges were added, then using UCSF Chimera software 1.19 to optimize the protein for further analysis.

#### 4.21.2 Ligands Preparation

Seventeen identified compounds in the HPLC‐ESI‐QTOF‐MS analysis of *A. halimus* and *S. fruticosa* extracts were selected for ligand preparation to target acetylcholinesterase (AChE) receptors. Their corresponding three‐dimensional (3D) chemical structures were retrieved from the PubChem database (https://pubchem.ncbi.nlm.nih.gov/) in SDF format and converted to PDB format into Open Babel GUI [37].

#### 4.21.3 Molecular Docking

Molecular docking was carried out using PyRx software. Ligand structures were imported into the platform via the integrated Open Babel module and subsequently converted into an AutoDock ligand. The acetylcholinesterase (AChE) protein structure was also converted into the AutoDock macromolecule. Docking simulations were performed using the Vina Wizard tool in PyRx. The grid box was centered at (*x* = 25.88, *y* = 62.5, *z* = 12.34) with dimensions of 22 × 22 × 22 Å to ensure appropriate coverage of the binding pocket.

To validate the docking protocol, a redocking procedure was conducted by extracting the native co‐crystallized ligand and re‐docking it into the active site under identical docking parameters. The resulting docked conformation was superimposed onto the crystallographic pose using UCSF Chimera, and the root mean square deviation (RMSD) was calculated. An RMSD value below 2.0 Å confirmed the reliability and reproducibility of the docking protocol. The docking results were exported in CSV format, and the ligand–protein interactions were further analyzed and visualized in both 2D and 3D using Discovery Studio Visualizer 2025 Client [37].

#### 4.21.4 Statistical Analysis

All experiments were performed in triplicate (*n* = 3), and results were expressed as mean ± standard deviation (M ± SD) using the Statistical Package for the Social Sciences (SPSS) software, version 26.0 (IBM, 2019). Data normality was assessed using the Shapiro–Wilk test, and all datasets satisfied the assumptions of normality (*p* > 0.05); therefore. Total phenolic, flavonoid, and tannin contents were analyzed using one‐way analysis of variance (ANOVA), followed by Tukey's post hoc test for multiple comparisons. Insecticidal activity was evaluated using log‐probit regression to estimate LC_50_ values along with their 95% confidence intervals. Statistical significance was set at *p* ≤ 0.05.

## Author Contributions

**Mounira Tabouri**: data curation, formal analysis, investigation, resources; writing – original draft. **Mustapha Djellouli**: conceptualization, methodology, software, supervision, validation, writing – review and editing. **Khadidja Belkheir**: validation, writing – review and editing. **Kamel Benotmane**: funding acquisition, investigation, visualization. **Meriem Dahoun**: funding acquisition, investigation. **Abdesselem Si Mohammed**: resources. **Saïd Nemmiche**: formal analysis, software. **Hamdi Bendif**: funding acquisition, writing – review and editing, supervision. **Elfalah Walid**: software, project administration, funding acquisition. **Boufahja Fehmi**: software, project administration, funding acquisition. **Alsalamah Sulaiman A**.: data curation, project administration. **Stefania Garzoli**: writing – review and editing, supervision. **Inmaculada Moscoso‐Ruiz**: formal analysis; validation. **Inés Cea‐Pavez**: supervision; validation. **Vito Verardo**: supervision; validation. All authors have read and agreed to the published version of the manuscript.

## Funding

This research did not receive any specific grant from funding agencies in the public, commercial, or not‐for‐profit sectors.

## Conflicts of Interest

The authors declare no conflicts of interest.

## Data Availability

Data will be made available on request.

## References

[1] FAO , “Global Information and Early Warning System on Food and Agriculture,” (GIEWS Country Brief, 2025).

[2] N. Bakroune , A. T. Ouamane , M. Boultif , F. Bettiche , and S. T. Torki , “Diversity of Cereal Pests (Wheat and Barley) Grown in arid Climate in Ziban Region (Provence of Biskra‐ Southeastern Algeria),” Studia UBB Biologia 69 (2024): 23–45.

[3] A. Ogreten , S. Eren , C. Kaya , et al., “Insecticidal Efficacy of Native Raw and Commercial Diatomaceous Earths Against *Tribolium confusum* DuVal (Coleoptera: Tenebrionidae) Under Different Environmental Conditions,” Journal of King Saud University – Science 35 (2023): 102827.

[4] D. Moutassem , T. Boubellouta , Y. Bellik , et al., “Insecticidal Activity of *Thymus pallescens* de Noë and *Cymbogon citratus* Essential Oils Against *Sitophilus zeamais* and *Tribolium castaneum* ,” Scientific Reports 14 (2024): 13951. 38886531 10.1038/s41598-024-64757-3 PMC11183130

[5] C. Hassaine , R. Aboura , A. Merzouk , and D. Benmansour , “Study of Halophytes Dispersion in the North‐West Region of Algeria,” Open Journal of Ecology 04 (2014): 628–640.

[6] M. Stanković , Z. Stojanović‐Radić , D. Jakovljević , N. Zlatić , M. Luković , and Z. Dajić‐Stevanović , “Coastal Halophytes: Potent Source of Bioactive Molecules From Saline Environment,” Plants 12 (2023): 1857. 37176915 10.3390/plants12091857 PMC10181147

[7] M. Hasanuzzaman , K. Nahar , M. M. Alam , et al., “Potential Use of Halophytes to Remediate Saline Soils,” BioMed Research International 2014 (2014): 589341. 25110683 10.1155/2014/589341 PMC4109415

[8] M. M. M. Soliman , A. A. Hassanein , and H. M. Abou‐Yousef , “Efficiency of Various Wild Plant Extracts Against the Cotton Aphid, *Aphis gossypii* Glov. (Aphididae: Homoptera),” Acta Phytopathologica et Entomologica Hungarica 40 (2005): 185–196.

[9] D. Saïdana , M. B. Halima‐Kamel , M. A. Mahjoub , D. Haouas , Z. Mighri , and A. N. Helal , “Insecticidal Activities of Tunisian Halophytic Plant Extracts Against Larvae and Adults of *Tribolium confusum′* ,” Tropicultura 25 (2007): 193–199.

[10] D. J. Walker , S. Lutts , M. Sánchez‐García , and E. Correal , “ *Atriplex halimus* L.: Its Biology and Uses,” Journal of Arid Environments 4 (2014): 111–121.

[11] H. Saleem , U. Khurshid , M. Sarfraz , et al., “A Comprehensive Phytochemical, Biological, Toxicological and Molecular Docking Evaluation of Suaeda fruticosa (L.) Forssk.: An Edible Halophyte Medicinal Plant,” Food and Chemical Toxicology 154 (2021): 112348. 34144099 10.1016/j.fct.2021.112348

[12] A. Ayaz , Q. Jamil , M. Hussain , et al., “Antioxidant and Gastroprotective Activity of *Suaeda fruticosa* Forssk. Ex J.F. Gmel,” Molecules 27 (2022): 4368. 35889240 10.3390/molecules27144368 PMC9322968

[13] M. Roubi , A. Elbouzidi , M. Dalli , et al., “Phytochemical, Antioxidant, and Anticancer Assessments Of Atriplex halimus Extracts: In Silico and In Vitro Studies,” Scientific African 22 (2023): e01959.

[14] R. Suthar and H. A. Solanki , “Phytochemical Screening of Halophytic Plant *Suaeda fruticosa* (L.) Forssk. Ex J.F. Gmel,” International Association of Biological and Computational Digest 1 (2022): 308–313.

[15] E. Bounouar , F. Missoun , N. O. Amari , et al., “Antidiabetic effect of *Atriplex halimus* Long and Short Term Treatment Against Streptozotocin Induced Diabetes in Rats,” Anales de Biologia 44 (2022): 21–30.

[16] N. Benhammou , F. A. Bekkara , and T. Kadifkova Panovska , “Antioxidant Activity of Methanolic Extracts and Some Bioactive Compounds of *Atriplex halimus* ,” Comptes Rendus Chimie 12 (2009): 1259–1266.

[17] A. S. Ould Kaddour , M. Bouzouina , and B. Lotmani , “Phenolics Contents and In‐Vitro Evaluation of the Antioxidant Effects of the Aerial Parts of Three Algerian *Atriplex halimus* L. Ecotypes,” Plant Archive 19 (2019): 1583–1592.

[18] A. Tadj , M. Achir , K. Souana , L. A. Abderrahim , M. Boussaid , and K. Taïbi , “Evaluation of the Phytochemical Composition, Antioxidant, Anti‐Inflammatory and Antihemolytic Activities of *Atriplex halimus* L. From the Region of Tiaret, Algeria,” Science and Health Sciences 5 (2024): e12637.

[19] N. Chekroun‐Bechlaghem , N. Belyagoubi‐Benhammou , L. Belyagoubi , et al., “Phytochemical Analysis and Antioxidant Activity of Tamarix africana, Arthrocnemum macrostachyum and Suaeda fruticosa, Three Halophyte Species From Algeria,” Plant Biosystems 153 (2019): 843–852.

[20] N. Gheraissa , A. E. Chemsa , N. Cherrada , et al., “Biochemical Profile and In Vitro Therapeutic Properties of Two Euhalophytes, Halocnemum strobilaceum Pall. and Suaeda fruticosa (L.) Forske., Grown in the Sabkha Ecosystem in the Algerian Sahara,” Molecules 28 (2023): 3580. 37110814 10.3390/molecules28083580 PMC10141351

[21] R. Quirantes‐Piné , J. Lozano‐Sánchez , M. Herrero , E. Ibáñez , A. Segura‐Carretero , and A. Fernández‐Gutiérrez , “HPLC–ESI–QTOF–MS as a Powerful Analytical Tool for Characterising Phenolic Compounds in Olive‐leaf Extracts,” Phytochemical Analysis 24 (2013): 213–223. 22987739 10.1002/pca.2401

[22] S. Kumar , A. Singh , and B. Kumar , “Identification and Characterization of Phenolics and Terpenoids From Ethanolic Extracts of *Phyllanthus* Species by HPLC‐ESI‐QTOF‐MS/MS,” Journal of Pharmaceutical Analysis 7 (2017): 214–222. 29404041 10.1016/j.jpha.2017.01.005 PMC5790687

[23] M. Clauser , S. Dall'Acqua , M. C. Loi , and G. Innocenti , “Phytochemical Investigation on Atriplex halimus L. From Sardinia,” Natural Product Research 27 (2013): 1940–1944. 23639103 10.1080/14786419.2013.793684

[24] V. Pereira , O. Figueira , and P. C. Castilho , “Flavonoids as insecticides in crop protection—A Review of Current Research and Future Prospects,” Plants 13 (2024): 776. 38592833 10.3390/plants13060776 PMC10975847

[25] I. Naboulsi , K. El Fakhouri , R. Lamzira , et al., “Insecticidal activities of *Atriplex halimus* L., *Salvia rosmarinus* Spenn. and *Cuminum cyminum* L. against *Dactylopius opuntiae* (Cockerell) Under Laboratory and Greenhouse Conditions,” Insects 13 (2022): 930. 36292878 10.3390/insects13100930 PMC9603841

[26] H. Soummane , M. Larhsini , K. Naamani , and J. Coll , “Studies of Larvicidal and Adulticidal Activities of Some Halophyte Plant Extracts Against *Ceratitis capitata* (Wiedemann),” Journal of Entomology 8 (2011): 548–556.

[27] P. Quézel and S. Santa , “Nouvelle Flore de l'Algérie et Des Régions Désertiques Méridionales,” (CNRS, 1962).

[28] A. Djidel , S. Daghbouche , A. Benrima , and Z. E. Djazouli , “Évaluation de l'activité Insecticide de l'extrait Aqueux Brut de la Fabacae Cytisus triflorus à L′égard de Tribolium castanium (Herbst, 1797) (Coleoptera: Tenebrionidae),” Revue Agrobiologia 8 (2018): 1093–1102.

[29] J. R. Shaikh and M. Patil , “Qualitative Tests for Preliminary Phytochemical Screening: An Overview,” International Journal of Chemical Studies 8 (2020): 603–608.

[30] L. Maheshwaran , L. Nadarajah , S. P. N. N. Senadeera , C. B. Ranaweera , A. K. Chandana , and R. N. Pathirana , “Phytochemical Testing Methodologies and Principles for Preliminary Screening/Qualitative Testing,” Asian Plant Research Journal 12 (2024): 11–38.

[31] L. Al Hawat and L. Alallan , “Estimation of Antioxidant and Hypolipidemic Activities of Extracts of Citrus xaurantium Leaves In Vitro,” Phytomedicine Plus 5 (2025): 100723.

[32] C. Elagdi , K. Bouaouda , R. Rahhal , et al., “Phenolic Compounds, Antioxidant and Antibacterial Activities of the Methanolic Extracts of Euphorbia resinifera and Euphorbia echinus,” Scientific African 21 (2023): 01779.

[33] H. H. El‐Kamali , “Effect of certain Medicinal Plants Extracts Against Storage Pest, *Tribolium castaneum* Herbst,” American‐Eurasian Journal of Sustainable Agriculture 3 (2009): 139–142.

[34] W. S. Abbott , “A Method of Computing the Effectiveness of an Insecticide,” Journal of Economic Entomology 18 (1925): 265–267.

[35] D. T. Finney , Probit Analysis, 3rd ed. (Cambridge University Press, 1971), 20–42.

[36] A. Rawat , O. Prakash , K. Nagarkoti , et al., “Chemical Profiling and Bioactivity Evaluation Of Thymol‐Rich Coleus aromaticus Benth. Essential Oil,” Medicinal Plant Biology 3 (2024): e007.

[37] B. Verma , H. Karakoti , R. Kumar , et al., “Phytochemical Screening and Evaluation of Pesticidal Efficacy in the Oleoresins of Globba sessiliflora Sims and In Silico Study,” Evidence‐Based Complementary and Alternative Medicine 2023 (2023): 5936513. 36636605 10.1155/2023/5936513 PMC9831701

